# Clinical efficacy of laparoscopic radical cystectomy with intracorporeal urinary diversion and an analysis of factors influencing complications

**DOI:** 10.3389/fonc.2025.1592406

**Published:** 2025-06-06

**Authors:** Hongzhi Fang, Huihua Ji, Changjian Shi, Benrui Zhou, Jie Xu, Yuli Luo, Yunfei Li

**Affiliations:** Department of Urology, Renmin Hospital, Hubei University of Medicine, Shiyan, Hubei, China

**Keywords:** bladder cancer, laparoscopy, urinary diversion, complications, risk factors

## Abstract

**Purpose:**

To explore the feasibility of combined laparoscopic radical cystectomy (LRC) and intracorporeal urinary diversion (ICUD) in the treatment of bladder cancer, as well as the influencing factors related to complications.

**Methods:**

A retrospective study was conducted on 116 bladder cancer patients who underwent LRC at our facility between January 2019 and December 2023. Based on the different urinary diversion methods, 78 patients received extracorporeal urinary diversion (ECUD), while 38 patients underwent intracorporeal urinary diversion (ICUD). The two groups were compared in terms of clinicopathologic data, perioperative outcomes, postoperative tumor control, complication rates, and their influencing factors.

**Results:**

No statistically significant differences were observed between the two groups in terms of median total operative time, hospital stay, perioperative transfusion rate, and short-term oncological outcomes. Compared to the ECUD group, the ICUD group experienced less intraoperative blood loss (200 ml vs. 350 ml) and an earlier start to postoperative liquid diet intake (4 days vs. 5 days) (p < 0.05). A total of 24 cases of ≥III grade complications occurred within 90 days postoperatively, with 20 cases in the ECUD group and 4 in the ICUD group. There was no significant difference in the incidence of ≥III grade complications between the two groups (p > 0.05). Sepsis was the most common major complication. Logistic regression analysis identified smoking history, diabetes, and intraoperative blood loss as independent risk factors for ≥III complications.

**Conclusions:**

ICUD is a secure and effective method with advantages such as improved postoperative bowel recovery, reduced intraoperative blood loss, and fewer overall postoperative complications. Furthermore, major complications are influenced by multiple risk factors and should be carefully considered during preoperative and postoperative management.

## Introduction

Bladder cancer ranks among the top ten most prevalent malignancies worldwide, constituting a major public health challenge due to its high incidence and mortality rates (1). Radical cystectomy (RC) remains the standard treatment for muscle-invasive bladder cancer (MIBC) and selected cases of high-risk non-muscle-invasive bladder cancer (NMIBC) (2). The significant surgical trauma, prolonged recovery time, and substantial complication rates associated with conventional open radical cystectomy (ORC) have driven the development of minimally invasive alternatives. Since its introduction in the late 20th century, laparoscopic radical cystectomy (LRC) has become an important surgical option, offering advantages including reduced intraoperative blood loss, improved visualization, and decreased postoperative pain (3–7). The subsequent adoption of robot-assisted laparoscopic technology (RARC) has further refined these procedures (8–10).

Urinary diversion represents a pivotal aspect of radical cystectomy, with surgical technique selection demonstrating strong associations with postoperative quality of life and complication risks. Emerging evidence suggests a progressive transition toward intracorporeal urinary diversion (ICUD) over extracorporeal methods (ECUD), particularly with robot-assisted ICUD gaining clinical preference due to its abbreviated learning curve and enhanced recovery profiles (10–13). While ICUD offers benefits like lower wound infection rates and reduced pain, it introduces challenges such as extended operative durations and technical complexity (14).Importantly, diversion methodology substantially impacts complication patterns, particularly for gastrointestinal dysfunction, urinary infections, anastomotic strictures, and metabolic disturbances (15). Given the limited comparative data on laparoscopic intracorporeal versus extracorporeal approaches, this study systematically evaluates their differences following LRC, focusing on perioperative outcomes, complications, and risk determinants.

## Materials and methods

### Patients

We collected 116 patients with bladder cancer who underwent LRC for bladder tumors at our facility from January 2019 to December 2023. Indications for surgery were (1) muscle-invasive bladder cancer (T2–4a, N0–x, M0) and (2) high-risk non-muscle-invasive bladder cancer, high-risk recurrent superficial bladder cancer, or extensive non-muscle-invasive bladder cancer refractory to transurethral resection of bladder tumor and bladder perfusion therapy. Exclusion criteria included distant tumor metastasis, severe cardiopulmonary insufficiency, and positive intraoperative urethral margins. The same physician with substantial laparoscopic surgery experience performed all surgeries. Our research obtained the endorsement of the ethics committee in our institution.

### Objectives of the study

The primary objective of this study was to evaluate early complications in bladder cancer patients within 90 days after *in vivo* urinary diversion. These complications were classified based on their type and severity according to the Clavien-Dindo grading system, and the associated risk factors were analyzed using logistic regression (16, 17). The secondary objective was to assess the efficacy and feasibility of this treatment, with a focus on indicators such as tumor recurrence and metastasis, duration of surgery, and blood loss.

### Statistical analysis

All statistical analyses were performed using R software version 4.4.0 and SPSS version 23.0. For continuous variables, t-tests were used, while categorical variables were assessed using chi-square tests. Kaplan-Meier survival curves were created with the survival package in R, and the Log-rank test was applied to compare the survival curves between the two groups to assess statistical significance. To analyze the factors associated with complications, all patients were included as study subjects, and logistic regression analysis was conducted. Continuous variables were categorized based on the median. The multivariate logistic regression model included covariates with a p-value of less than 0.1 in the univariate analysis. A p-value of less than 0.05 was considered statistically significant in this detailed investigation.

### Surgical techniques

In the selection of surgical approaches for this study, we took into account factors such as the surgeon’s experience, the patient’s comorbidities, tumor characteristics, and personal preferences to determine whether ICUD or ECUD would be performed.

Following endotracheal intubation under general anesthesia, male patients were positioned supine while female patients were placed in the lithotomy position. A transperitoneal approach was adopted with the camera port established 3 fingerbreadths superior to the umbilical margin. Under laparoscopic guidance, 12-mm trocars were inserted bilaterally at the lateral borders of the rectus abdominis muscles 2 fingerbreadths inferior to the umbilicus. Additional 5-mm trocars were placed 3 cm medial to the anterior superior iliac spines bilaterally, with an auxiliary 5-mm port positioned at the right paraumbilical region for urinary diversion. The cystectomy procedure, standardized lymphadenectomy, and extracorporeal urinary diversion (ECUD) were performed as previously described in our published protocol (18).

Orthotopic neobladder reconstruction was accomplished using the Studer technique. A 45-cm ileal segment was isolated 15 cm proximal to the ileocecal junction using slow-mode ultrasonic dissection. Bowel continuity was restored through a functional end-to-end anastomosis using 60-mm and 45-mm linear staplers followed by transverse closure with a 60-mm blue cartridge. The efferent limb was secured to the urethral stump 10 cm distal to the afferent limb, with a 10-mm enterotomy created for urethro-ileal anastomosis. Preserving 10 cm of the proximal afferent limb intact, the remaining bowel segment was detubularized.

**Figure 1:**
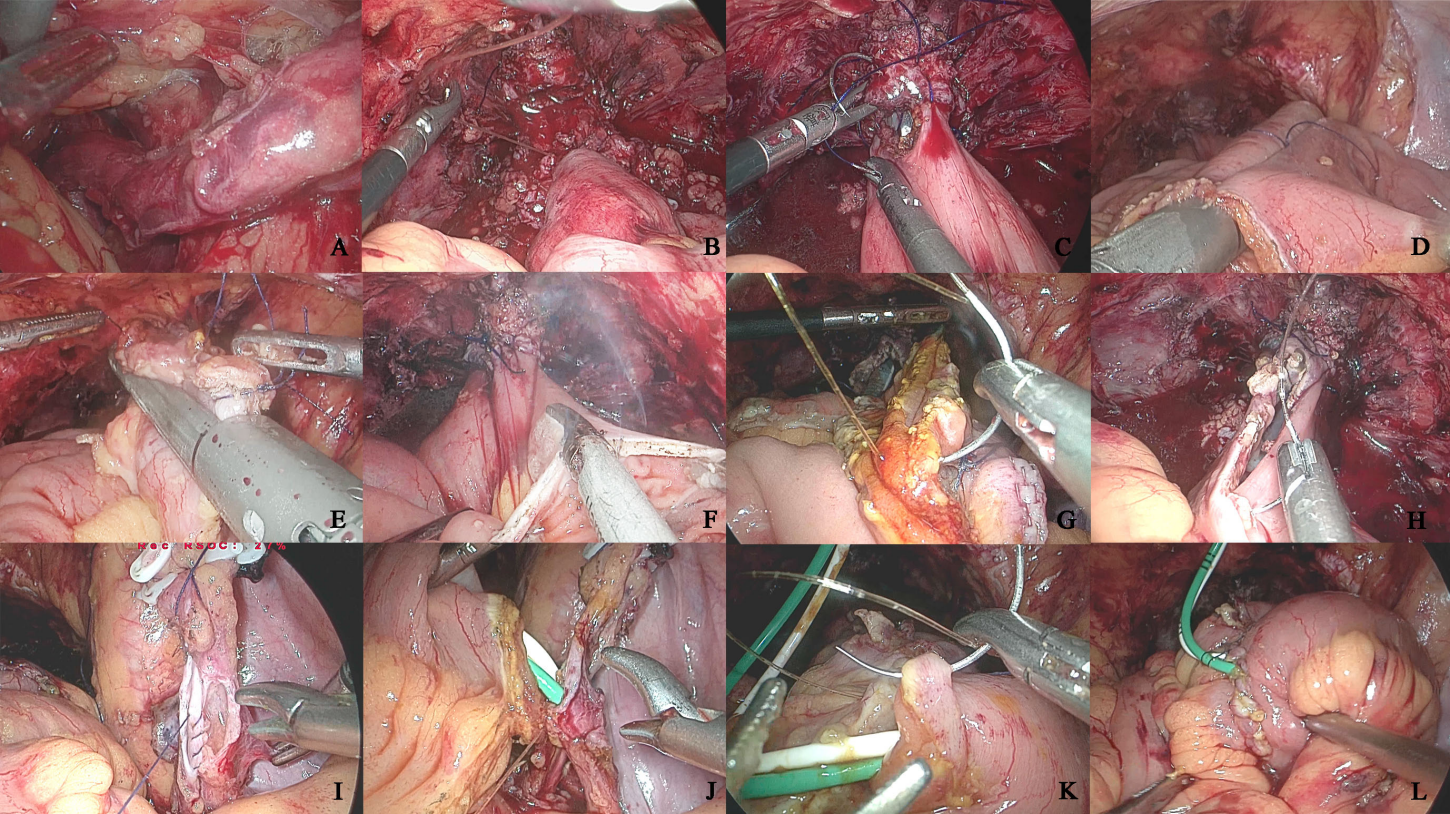
The key operational procedures of orthotopic neobladder construction intracorporeal. **(A)** Left ureteral retroperitoneal tunnel; **(B)** Anterior rectus fascia fixation of the intestine; **(C)** Intestinal-urethral anastomosis; **(D)** Longitudinal intestinal anastomosis; **(E)** Intestinal anastomosis; **(F)** Bowel detubularization; **(G)** Posterior wall anastomosis of the neobladder; **(H)** Anterior wall anterior half anastomosis of the neobladder; **(I)** Ureteral Wallance anastomosis; **(J)** Intestinal-ureteral anastomosis; **(K)** Anterior wall anastomosis of the neobladder; **(L)** Neobladder leakage test.

Posterior wall reconstruction was performed using continuous 3–0 Monocryl barbed suture in a U-shaped configuration, while the anterior wall was closed with 4–0 Monocryl barbed suture in a double-layered continuous fashion. Bilateral ureteroenteric anastomoses were completed using the Wallace technique with 5–0 Monocryl absorbable sutures. The 6 Fr Ruibang single-J stents were percutaneously inserted through the neobladder’s anteroinferior wall into both ureters and the afferent limb. Ureteroileal anastomoses were reinforced with 4–0 Monocryl barbed sutures, followed by final closure of the anterior bladder wall incorporating stent fixation using 3–0 barbed suture. A 20 Fr double-lumen catheter was indwelled after confirming watertight closure with 50 ml methylene blue-dyed saline irrigation. Uterine and adnexal preservation was maintained in female patients. Specimen extraction was achieved through extended pararectal Trocar incisions in males versus anterior vaginal wall incision in females. Critical procedural steps are illustrated in **Figures 1A–L** (**Figure 1**).

## Results

A total of 116 individuals underwent successful surgeries. The preoperative general information and clinicopathologic features of patients in the ECUD and ICUD groups are shown in **Table 1**. Of these patients, 97 (83.6%) were men and 19 (16.4%) were women. The gender differences between the two groups were significant (p < 0.05). However, no significant differences were observed between the groups in terms of age, BMI, smoking history, history of abdominal surgery, history of TURBT, prior radiation, hypertension, diabetes mellitus, coronary artery disease, ECOG scores, ASA scores, preoperative staging, and preoperative grading (p > 0.05).

**Table 1:** Demographics and Pre-operative Clinical Characteristics of Patients Undergoing LRC with ECUD and ICUD.

Variables	All patients (n = 116)	ECUD (n = 78)	ICUD (n = 38)	*P*
Age(years)	64.9 ± 8.8	64.0 ± 8.6	66.6 ± 9.2	0.145
Sex				0.044
Male	97 (83.6)	69 (88.5)	28 (73.7)	
Female	19 (16.4)	9 (11.5)	10 (26.3)	
BMI(kg/m^2^)	22.7 ± 2.8	22.9 ± 2.97	22.4 ± 2.5	0.445
Smoking status	56 (48.3)	40 (51.3)	16 (42.1)	0.353
Previous abdominal surgery	17 (14.7)	10 (12.8)	7 (18.4)	0.423
Prior TURBT	29 (25.0)	23 (29.5)	6 (15.8)	0.110
Neoadjuvant chemotherapy	20 (17.2)	14 (17.9)	6 (15.8)	0.773
Hypertension	35 (30.2)	23 (29.5)	12 (31.6)	0.818
Diabetes	18 (15.5)	14 (17.9)	4 (10.5)	0.300
CAD	21 (18.1)	16 (20.5)	5 (13.2)	0.334
ECOG				0.203
0	85 (73.3)	60 (76.9)	25 (65.8)	
≥1	31 (26.7)	18 (23.1)	13 (34.2)	
ASA score				0.438
1	16 (13.8)	11 (14.1)	5 (13.2)	
2	63 (54.3)	45 (57.7)	18 (47.4)	
3	35 (30.2)	20 (25.6)	15 (39.5)	
4	2 (1.7)	2 (2.6)	0 (0.0)	
Preoperative T stage				>0.99
Ta	5 (4.3)	3 (3.8)	2 (5.3)	
T1	20 (17.2)	14 (17.9)	6 (15.8)	
T2	72 (62.1)	48 (61.5)	24 (63.2)	
T3	16 (13.8)	11 (14.1)	5 (13.2)	
T4	3 (2.6)	2 (2.6)	1 (2.6)	
Preoperative grade				0.745
Low	39 (33.6)	27 (34.6)	12 (31.6)	
High	77 (66.4)	51 (65.4)	26 (68.4)	

Data expressed in medians (range) or n (%). BMI, Body mass index; ASA, American Society of Anesthesiologists (classification); ECOG, Eastern Cooperative Oncology Group; TURBT, Transurethral resection of bladder tumor; CAD, Coronary artery disease; ECUD, Extracorporeal urinary diversion; ICUD, Intracorporeal urinary diversion.

**Figure 2:**
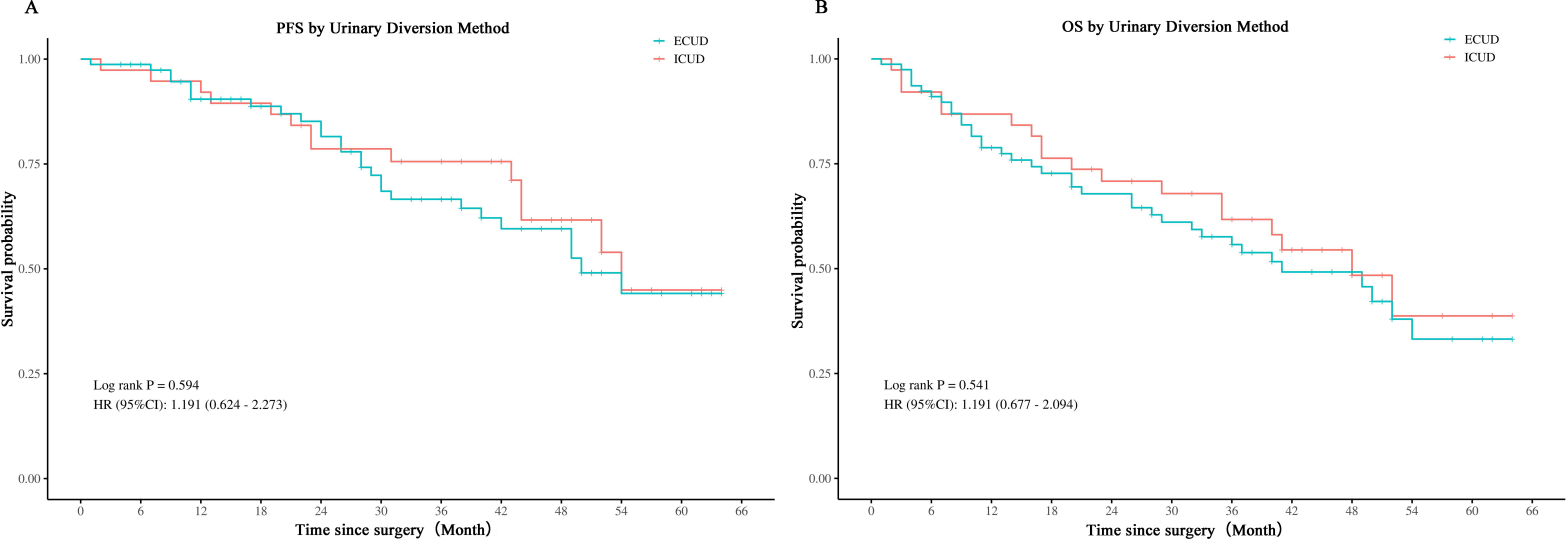
Comparison of PFS and OS in LRC with ECUD and ICUD. ECUD, Extracorporeal urinary diversion; ICUD, Intracorporeal urinary diversion; PFS, Progression-free survival; OS, Overall survival; LRC, Laparoscopic radical cystectomy.

The whole cohort’s perioperative and histologic results, broken down by type of urinary diversion, are shown in **Table 2**. The length of hospital stay, time to void, median surgical duration, and number of lymph nodes excised did not significantly differ between the two groups. The median total operating time for the ICUD group was 385 minutes, whereas the median for the ECUD group was 355 minutes. Regarding the type of urine diversion and intraoperative blood loss as well as the start of a fluid diet, there were significant differences between the two groups. Among the 78 ECUD procedures, 68 (87.2%) involved ileal conduits and 10 (12.8%) involved *in situ* neobladders. In contrast, among the 38 ICUD procedures, 26 (68.4%) involved ileal conduits and 12 (31.6%) involved *in situ* neobladders. In the ECUD group, the median estimated blood loss was 350 ml, while in the ICUD group, it was 200 ml (*p* < 0.001). Nonetheless, there was no discernible difference in the intraoperative transfusion rate between the two groups. Initiating a fluid diet took a median of 5 days for the ECUD group and 4 days for the ICUD group (*p* < 0.001). Postoperative staging, grading, margin positivity, lymph node positivity, and incidental prostate cancer did not significantly differ between the two groups. The ECUD collective median follow-up period was 28.5 months, whereas the ICUD collective was 41 months. Thirty-seven patients (47.4%) in the ECUD and eighteen patients (47.4%) in the ICUD experienced tumor progression. The corresponding median progression-free survival (PFS) was 48 months and 41 months. Between the two groups, no statistically significant disparity was found. A total of 27 patients (34.6%) in the ECUD group and 14 patients (36.8%) in the ICUD group passed away. There was no statistically significant difference (*p* > 0.05) between the ECUD and ICUD median overall survival (OS), which was 50 months and 54months, respectively (**Figure 2**).

**Table 2:** Perioperative outcomes of patients undergoing LRC with ECUD and ICUD.

Variables	All patients (n = 116)	ECUD (n = 78)	ICUD (n = 38)	P
Total time (min)	360 (315, 431.3)	355 (300, 422.5)	385 (333.8, 447.3)	0.072
Diversion time (min)	95 (85,115)	90 (85,100)	117.5 (90,143.8)	<.001
LOS (days)	16 (12, 20)	15 (12, 19)	17.5 (13.3, 21)	0.169
EBL (ml)	300 (187.5, 400)	350 (200, 487.5)	200 (100, 300)	<.001
Time of flatus (days)	3 (2, 3)	3 (2, 3.8)	3 (2, 3)	0.115
Time of intake of liquid diet (days)	4 (3, 5)	5 (4, 6)	4 (3, 4)	<.001
LNY	13 (9, 18)	12.5 (9, 19)	14 (11, 16)	0.972
IT	31 (26.7)	25 (32.1)	6 (15.8)	0.063
Diversion type				0.016
Ileal conduit	94 (81)	68 (87.2)	26 (68.4)	
Neobladder	22 (19)	10 (12.8)	12 (31.6)	
Postoperative pT stage				0.499
Ta	5 (4.3)	3 (3.8)	2 (5.3)	
T1	22 (19)	12 (15.4)	10 (26.3)	
T2	48 (41.4)	32 (41)	16 (42.1)	
T3	31 (26.7)	24 (30.8)	7 (18.4)	
T4	10 (8.6)	7 (9.0)	3 (7.9)	
Postoperative grade				0.817
Low	23 (19.8)	15 (19.2)	8 (21.1)	
High	93 (80.2)	63 (80.8)	30 (79)	
lymph node positive	16 (13.8)	13 (16.7)	3 (7.9)	0.198
PSM	4 (3.4)	3 (3.8)	1 (2.6)	>0.99
Incidental prostate cancer	11 (9.5)	8 (10.3)	3 (7.9)	0.944
Tumor progression	55 (47.4)	37 (47.4)	18 (47.4)	0.995
Tumor death	41 (35.3)	27 (34.6)	14 (36.8)	0.814
Follow−up time (months)	32.5 (16.8, 47.3)	28.5 (13, 46)	41 (23.8, 48.8)	0.047
PFS (months)	48 (37,59)	41 (27.8,54.2)	48 (36.4,59.6)	0.541
OS (months)	54 (48.1,59.9)	50 (38,62)	54 (39.6,68.4)	0.594

Data expressed in medians (range) or n (%). LOS, Length of stay; EBL, Estimated blood loss; IT, Intraoperative transfusion; LNY, Lymph node yield; PSM, Positive surgical margin; PFS, Progression-free survival; OS, Overall survival; ECUD, Extracorporeal urinary diversion; ICUD, Intracorporeal urinary diversion.

**Figure 3:**
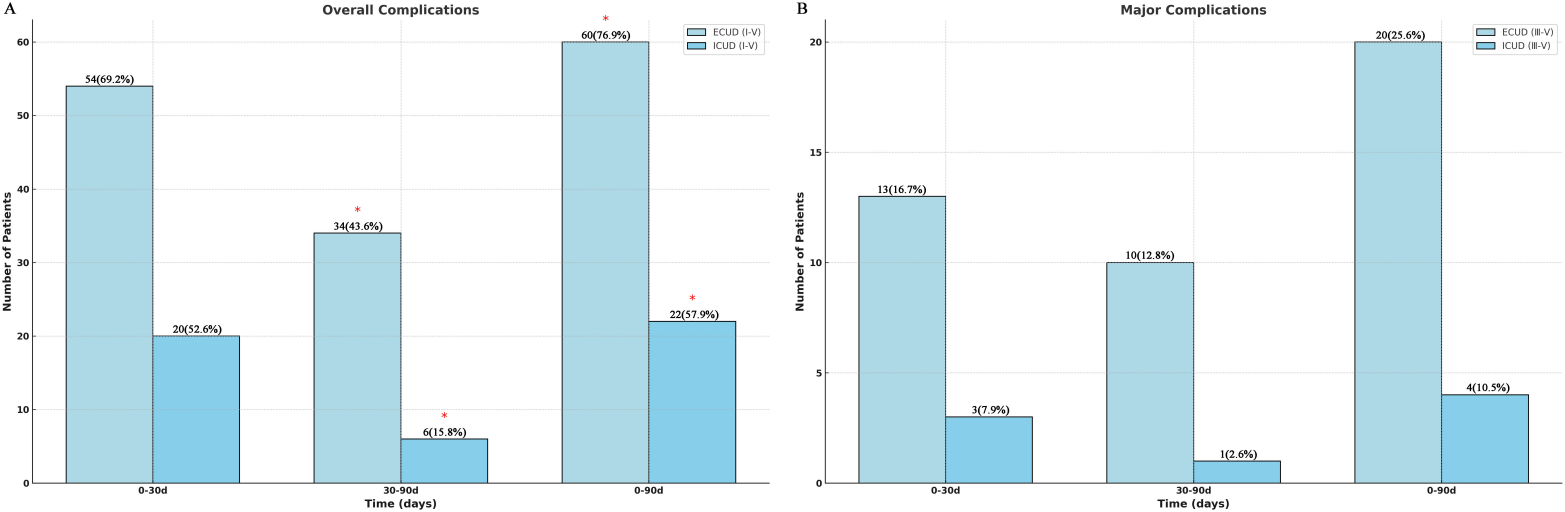
Clavien-Dindo classification of total complications (I-V) and major complications (III-V).

Regarding complications during 0–30 days, there was no significant difference in the overall Clavien-Dindo grades I–V and grade ≥ III incidences of complications within the ECUD and ICUD (**Figure 3A**) (p > 0.05). **Table 3** presents the detailed manifestations of complications in both groups. Additionally, **Supplementary Table S1** covers the treatment protocols for these complications. Fifteen cases (19.2%) within the ECUD and four cases (10.5%) within the ICUD experienced paralytic intestinal obstruction; all of these cases were categorized as grade II. The most common complications were hypoproteinemia and hypokalemia, both classified as grade I.

**Table 3:** Complication profiles of patients within 0–30 days and 31–90 days after undergoing LRC with ECUD and ICUD.

Time	Complication	All patients (n = 116)	ECUD (n = 78)	ICUD (n = 38)	Clavien grade
0–30 Days	Paralytic ileus	19 (16.4)	15 (19.2)	4 (10.5)	II
	Pulmonary infection	6 (5.2)	4 (5.1)	2 (5.3)	II
	Hydronephrosis	3 (2.6)	2 (2.6)	1 (2.6)	III
	Septicemia	4(3.4)	3 (3.8)	1 (2.6)	IV
	DVT	8 (6.9)	6 (7.7)	2 (5.3)	II
	Ileostomy bleeding	3 (2.6)	2 (2.6)	1 (2.6)	III
	UTI	12 (10.3)	8 (10.3)	4 (10.5)	II
	Lymphatic leakage	3 (2.6)	1 (1.3)	2 (5.3)	II
	Abdominal infection	5 (4.3)	2 (2.6)	3 (7.9)	II
	Wound dehiscence	3 (2.6)	3 (3.8)	0	III
	Incarcerated hernia	1 (0.9)	1 (1.3)	0	III
	Urinary fistula	2 (1.7)	2 (2.6)	0	III
	Hypokalemia	25 (21.6)	14 (17.9)	11 (29)	I
	FUO	9 (7.8)	6 (7.7)	3 (7.9)	II
	Hypoproteinemia	35 (30.2)	24 (30.8)	11 (29)	II
	Anemia	26 (22.4)	20 (25.6)	6 (15.8)	I
	Blood transfusion	7 (6.0)	6 (7.7)	1 (2.6)	II
	Renal insufficiency	19 (16.4)	13 (16.7)	6 (15.8)	I
31–90 Days	Paralytic ileus	6 (5.2)	6 (7.7)	0	II
	Hydronephrosis	4 (3.4)	4 (5.1)	0	III
	Septicemia	4 (3.4)	4 (5.1)	0	IV
	DVT	4 (3.4)	4 (5.1)	0	II
	UTI	19 (16.4)	17 (21.8)	2 (5.3)	II
	Abdominal infection	2 (1.7)	1 (1.3)	1 (2.6)	II
	Anastomotic stricture	1 (0.9)	1 (1.3)	0	III
	Intestinal fistula	1 (0.9)	1 (1.3)	0	III
	Hypokalemia	4 (3.4)	4 (5.1)	0	II
	Hypoproteinemia	5 (4.3)	3 (3.8)	2 (5.3)	II
	Death	2 (1.7)	1 (1.3)	1 (2.6)	V

DVT, Deep venous thrombosis; FUO, Fever of unknown origin; UTI, Urinary tract infection; ECUD, Extracorporeal urinary diversion; ICUD, Intracorporeal urinary diversion.

The ICUD group experienced significantly fewer total grade I–V complications (6 vs. 34 cases) than the ECUD group (*p* < 0.05) in terms of complications between 30–90 days (**Figure 3B**). The rate of grade ≥ III complications, however, the discrepancy was not significant (P > 0.05). Late complication incidence is relatively low, but urinary tract infections are more common, with 17 cases (21.8%) in the ECUD and 2 cases (5.3%) in the ICUD, both classified as grade II. Furthermore, 11 patients (9.5%) developed grade ≥ III sequelae, the most frequent of which were hydronephrosis and sepsis. One patient in each group died at this stage. Overall, a total of 24 patients developed ≥III grade complications within 90 days postoperatively, with 16 cases occurring within 0–30 days and 8 cases occurring within 30–90 days. Based on the group, 20 cases occurred in the ECUD group and 4 cases in the ICUD group. No significant difference in the incidence of ≥III grade complications was observed between the two groups (p > 0.05).

Continuous variables were grouped according to their median values (**Supplementary Table S2**), and single and multivariate logistic regression models were employed to identify risk factors for major complications (**Table 4**). Univariate logistic regression analysis indicates that major complications were significantly correlated with smoking history (*p* = 0.047, OR = 2.60, 95% CI: 1.01–6.68), diabetes mellitus (*p* = 0.002, OR = 5.53, 95% CI: 1.89–16.22), and intraoperative hemorrhage (*p* = 0.009, OR = 4.15, 95% CI: 1.43–12.04). Additionally, smoking history (*p* = 0.045, OR = 2.90, 95% CI: 1.02–8.21), diabetes mellitus (*p* = 0.039, OR = 3.73, 95% CI: 1.07–13.06), and intraoperative bleeding (*p* = 0.035, OR = 3.53, 95% CI: 1.10–11.36) all got validated as separate risk variables by multivariate logistic regression analysis. Other variables, such as gender and coronary heart disease, were not statistically significant.

**Table 4:** Logistic regression analysis on the risk factors of Clavien-Dindo Grade 3 and above complications in patients after surgery.

Variables	Univariate		Multivariate *P* OR (95%CI)	
	*P*	OR (95%CI)	*P*	OR (95%CI)
Sex	0.566	0.68 (0.18 ~ 2.55)		
Previous abdominal surgery	0.341	1.75 (0.55 ~ 5.58)		
Smoking status	0.047	2.60 (1.01 ~ 6.68)	0.045	2.90 (1.02 ~ 8.21)
Prior TURBT	>0.99	1.00 (0.35 ~ 2.82)		
Neoadjuvant chemotherapy	0.209	0.37 (0.08 ~ 1.74)		
Hypertension	0.904	0.94 (0.35 ~ 2.52)		
Diabetes	0.002	5.53 (1.89 ~ 16.22)	0.039	3.73 (1.07 ~ 13.06)
CAD	0.035	3.04 (1.08 ~ 8.53)	0.170	2.39 (0.69 ~ 8.32)
ECOG	0.466	0.67 (0.23 ~ 1.98)		
ASA	0.865	1.09 (0.42 ~ 2.83)		
IT	0.413	1.50 (0.57 ~ 3.96)		
Diversion type	0.793	1.16 (0.38 ~ 3.55)		
Method of diversion	0.068	0.34 (0.11 ~ 1.08)	0.470	0.63 (0.18 ~ 2.22)
Age	0.468	1.40 (0.56 ~ 3.47)		
Operative time	0.862	1.08 (0.44 ~ 2.67)		
LOS	0.277	1.67 (0.66 ~ 4.19)		
EBL	0.009	4.15 (1.43 ~ 12.04)	0.035	3.53 (1.10 ~ 11.36)
Time of flatus	0.242	1.79 (0.68 ~ 4.73)		
Liquid diet duration	0.218	2.08 (0.65 ~ 6.65)		
BMI	0.647	1.23 (0.50 ~ 3.04)		

BMI, Body mass index; TURBT, Transurethral resection of bladder tumor; CAD, Coronary artery disease; ASA, American Society of Anesthesiologists (classification); ECOG, Eastern Cooperative Oncology Group; LOS, Length of stay; EBL, Estimated blood loss; IT, Intraoperative transfusion.

## Discussion

Since ICUD technology was first introduced by Gill et al. (19) in 2000, its primary application has been limited to high-level medical centers. The high technical complexity and the stringent experience required for surgeons have posed significant challenges to its widespread adoption. In recent years, advancements in robotic surgical systems and improvements in laparoscopic techniques have significantly shortened the learning curve for ICUD (20, 21). These technological advancements have enabled more medical centers to adopt ICUD as a viable treatment option. This study focused on analyzing perioperative outcomes, postoperative complications, and risk factors in patients undergoing ICUD and ECUD following laparoscopic cystectomy. At our hospital, the same surgeon carried out every surgery, guaranteeing a controlled study setting for a thorough examination of this intricate problem.

Hussein et al. (10) examined urine diversion after robot-assisted radical bladder removal intracorporeal and extracorporeal in a large multi-institutional retrospective research study. Among 1,094 patients, intracorporeal diversion was associated with a shorter operative time (357 min vs. 400 min), reduced blood loss (300 ml vs. 350 ml), and a lower transfusion rate (4% vs. 19%) (all *p* < 0.001). In the present study, intraoperative blood loss was considerably lower in the ICUD compared to the ECUD (200 ml vs. 350 ml, *p* < 0.05), consistent with the previous findings. Moreover, in our study, the total operative time in the ICUD was longer, and the diversion time was also longer (117.5 min vs. 90 min, *p* < 0.001). This could be attributed to the more complex nature of in - vivo urinary diversion procedures. Secondly, there was a higher number of in - situ neobladder procedures in the ICUD. Since this procedure requires precise vesicourethral anastomosis - a time - consuming step that demands intraoperative stability, it most likely extended the operative duration.

According to the study’s findings, the median time to start the fluid diet in the ICUD group was earlier than that in the ECUD group (4 days vs. 5 days, p < 0.001). While the total length of hospitalization was marginally more time in the ICUD group, the median time to extubation was comparable. These findings align with a study by Hussein et al. (22), which reported a longer hospitalization duration for ICUD patients (9 days vs. 8 days, *p* < 0.01). In contrast, Wang et al. (23) reported different findings, showing an earlier median time to first flatus in the ICUD (3 days vs. 5 days for ECUD) and a shorter postoperative hospitalization duration (11 days for ICUD vs. 17 days for ECUD). However, the median time to fluid diet intake was consistent with our study (4 days vs. 5 days, *p* < 0.05). Disparities in surgical skill and accuracy among surgical teams conducting urine diversion could be the cause of the disparities in hospitalization time and time to first flatus in our study. These differences, however, stayed within a reasonable range. According to an analysis by An et al. (24), ICUD significantly decreased the probability of blood transfusion (RR: 0.40; 95% CI: 0.24–0.68) and decreased intraoperative blood loss by 64.12 ml (95% CI: -100.95 to -27.29). In a comparative analysis of 174 patients undergoing robotic-assisted cystectomy, Fu et al. (25) found that, on average, ECUD patients needed substantially more blood transfusions than ICUD patients (1.0 vs. 0.5, *p* < 0.05). Each study had a different rate of perioperative blood transfusions. Our study found that although the ICUD experienced much less intraoperative blood loss than the ECUD (200 ml vs. 350 ml, *p* < 0.05), there was no significant difference in the blood transfusion rate (15.8% vs. 31.1%, *p* = 0.063). This may be because transfusion decisions consider vital signs, the surgical procedure, and strict transfusion criteria, rather than relying solely on the volume of blood loss.

There aren’t many research studies comparing the oncological outcomes of ICUD vs ECUD. The number of lymph nodes resected, positive tumor margins, and postoperative pathological stage did not significantly differ between ICUD and ECUD in this investigation. Furthermore, there were no distinctions in the median PFS or OS between the two groups, according to the Kaplan-Meier curves. These outcomes are in line with those of Ham et al. (26) and Kanno et al. (27), indicating that ICUD offers tumor control effectiveness on par with ECUD. Although ICUD and ECUD show similar outcomes, the choice of the appropriate surgical approach should consider individual patient factors, postoperative recovery, and quality of life. Future studies may further explore their comprehensive effects on long-term patient health and quality of life.

Within 30–90 days after surgery, Mazzone et al. (28) found no discernible difference in the ICUD and ECUD groups’ complication rates (Clavien-Dindo ≥ grade 2) (35% vs. 43%, *p* = 0.2). In a similar vein, our research revealed that over the long run (30–90 days), the ICUD team experienced fewer overall grade I–V complications than the ECUD team. Grade ≥ III complications, however, did not vary significantly. This may be explained by intracorporeal urinary diversion better preserving physiological structure and function, thereby reducing the risk of intestinal adhesions, obstruction, and urinary tract complications. A total of 24 patients (20.7%) experienced high-grade complications within 90 days, which remained within acceptable limits. Early complications primarily involved hypoproteinemia and hypokalemia, likely related to postoperative gastrointestinal dysfunction, impaired nutrient absorption, and surgical stress. The most frequent late consequence was urinary tract infection, which is probably brought on by the extracorporeal channel construction’s modification of urinary tract anatomy and function, which raises the chance of urine reflux. This finding aligns with Azzouni et al. (29), who reported urinary tract infection as the most common complication, accounting for 31% of all cases.

In this research, univariate and multivariate logistic regression examines identified smoking history, diabetes mellitus, and intraoperative bleeding as independent risk factors for high-level postoperative complications. Targeted perioperative interventions addressing these risk factors could decrease the incidence of postoperative complications and enhance patients’ postoperative recovery. Although coronary artery disease and urinary diversion method showed trends toward meaning in univariate analysis, they were not significant in multivariate analysis. This may be due to their effects being moderated or masked by more dominant variables. This indicates that these factors may still warrant attention in specific subgroups or clinical scenarios.

## Limitations

To begin, all surgical interventions were conducted by a single highly skilled surgeon. Although this approach ensured methodological consistency, it may reduce the broader relevance of the findings for practitioners with varying levels of expertise. Additionally, an uneven distribution of participants and urinary diversion methods was observed between the ICUD and ECUD cohorts. Such disparities could compromise statistical reliability, obscure a precise evaluation of the two techniques’ efficacy, and potentially obscure the detection of infrequent adverse events. Furthermore, variations in follow-up duration across groups might introduce bias when interpreting long-term results, including delayed complications and survival rates. Another limitation was the absence of patient-reported quality-of-life metrics, particularly concerning sustained psychosocial adjustment and functional rehabilitation following *in vivo* diversion. To overcome these constraints, future research should involve multi-institutional cooperation, larger sample sizes, uniform follow-up periods, and the incorporation of patient-centered outcome measures.

## Conclusions

ICUD is a safe and effective method of urinary diversion, offering advantages over ECUD, including less intraoperative blood loss, faster gastrointestinal recovery, and lower postoperative complication rates. This study indicates that ICUD shows superior early postoperative recovery and complication control compared to ECUD. Smoking history, diabetes, and intraoperative blood loss were identified as independent risk factors for major complications and should be prioritized in preoperative and postoperative management.

## Data Availability

The original contributions presented in the study are included in the article/**Supplementary Material**. Further inquiries can be directed to the corresponding author.
