# Supplementary material for: Clinical efficacy of laparoscopic radical cystectomy with intracorporeal urinary diversion and an analysis of factors influencing complications
Source: Front Oncol. 2025 Jun 6;15:1592406. doi: 10.3389/fonc.2025.1592406 (PMC12178858; doi:10.3389/fonc.2025.1592406)
Supplement: Supplementary file 1 [file Table1.docx]

**Table S1:** **Management Approaches for Complications**

| **Time** | **Complication** | **Treatment** |
| --- | --- | --- |
| 0-30 Days | Paralytic ileus | Conservative |
|  | Pulmonary infection | Antibiotics |
|  | Hydronephrosis | Nephrostomy or ureteral stent placement |
|  | Septicemia | transferred to the ICU |
|  | DVT | Anticoagulation |
|  | Ileostomy bleeding | Surgical treatment |
|  | UTI | Antibiotics |
|  | Lymphatic leakage | Conservative |
|  | Abdominal infection | Antibiotics |
|  | Wound dehiscence | Secondary debridement and suturing |
|  | Incarcerated hernia | Surgical treatment |
|  | Urinary fistula | Nephrostomy or ureteral stent placement |
|  | Hypokalemia | Potassium supplementation |
|  | FUO | Antipyretics plus antibiotics |
|  | Hypoproteinemia | Human albumin injection |
|  | Anemia | Observation |
|  | Blood transfusion | Transfusion |
|  | Renal insufficiency | Observation |
| 31-90 Days | Paralytic ileus | Conservative |
|  | Hydronephrosis | Nephrostomy or ureteral stent placement |
|  | Septicemia | transferred to the ICU |
|  | DVT | Anticoagulation |
|  | UTI | Antibiotics |
|  | Abdominal infection | Antibiotics |
|  | Anastomotic stricture | Nephrostomy or ureteral stent placement |
|  | Intestinal fistula | Surgical treatment |
|  | Hypokalemia | Potassium supplementation |
|  | Hypoproteinemia | Human albumin injection |
|  | Death | - |

**Table S2. Variable assignment for univariate and multivariate logistic regression**

| **Factors** | **Variables** | **Assignment** |
| --- | --- | --- |
| major complications | Y | Yes=1，no=0 |
| Sex | X1 | Male=1，Female=2 |
| Previous abdominal surgery | X2 | Yes=1，no =0 |
| Smoking status | X3 | Yes=1，no =0 |
| Prior TURBT | X4 | Yes=1，no =0 |
| Neoadjuvant chemotherapy | X5 | Yes=1，no =0 |
| Hypertension | X6 | Yes=1，no =0 |
| Diabetes | X7 | Yes=1，no =0 |
| CAD | X8 | Yes=1，no =0 |
| ECOG | X9 | 1=1，0=0 |
| ASA | X10 | ＜3=1，≥3=2 |
| IT | X11 | Yes=1，No=0 |
| Diversion type | X12 | Ileal conduit=1，Neobladder =2 |
| Method of diversion | X13 | ECUD=1, ICUD=2 |
| Age（years） | X14 | ＜66=1，≥66=2 |
| Operative time（min） | X15 | ＜360=1，≥360=2 |
| LOS（days） | X16 | ＜16=1，≥16=2 |
| EBL（ml） | X17 | ＜300=1，≥300=2 |
| Time of flatus（days） | X18 | ＜3=1，≥3=2 |
| Liquid diet duration（days） | X19 | ＜4=1，≥4=2 |
| BMI（kg/m^2^） | X20 | ＜22.55=1，≥22.55=2 |
